# Comprehensive MicroRNA Expression Profile of the Mammary Gland in Lactating Dairy Cows With Extremely Different Milk Protein and Fat Percentages

**DOI:** 10.3389/fgene.2020.548268

**Published:** 2020-12-03

**Authors:** Xiaogang Cui, Shengli Zhang, Qin Zhang, Xiangyu Guo, Changxin Wu, Mingze Yao, Dongxiao Sun

**Affiliations:** ^1^Key Laboratory of Animal Genetics, Breeding and Reproduction of Ministry of Agriculture and Rural Affairs, National Engineering Laboratory of Animal Breeding, College of Animal Science and Technology, China Agricultural University, Beijing, China; ^2^Key Lab of Medical Molecular Cell Biology of Shanxi Province, Institutes of Biomedical Sciences, Shanxi University, Taiyuan, China; ^3^Center for Quantitative Genetics and Genomics, Aarhus University, Tjele, Denmark

**Keywords:** mammary gland, mRNA, miRNA, RNA-seq, dairy cattle

## Abstract

A total of 31 differentially expressed genes in the mammary glands were identified in our previous study using RNA sequencing (RNA-Seq), for lactating cows with extremely high and low milk protein and fat percentages. To determine the regulation of milk composition traits, we herein investigated the expression profiles of microRNA (miRNA) using small RNA sequencing based on the same samples as in the previous RNA-Seq experiment. A total of 497 known miRNAs (miRBase, release 22.1) and 49 novel miRNAs among the reads were identified. Among these miRNAs, 71 were found differentially expressed between the high and low groups (*p* < 0.05, *q* < 0.05). Furthermore, 21 of the differentially expressed genes reported in our previous RNA-Seq study were predicted as target genes for some of the 71 miRNAs. Gene ontology and KEGG pathway analyses showed that these targets were enriched for functions such as metabolism of protein and fat, and development of mammary gland, which indicating the critical role of these miRNAs in regulating the formation of milk protein and fat. With dual luciferase report assay, we further validated the regulatory role of 7 differentially expressed miRNAs through interaction with the specific sequences in 3′UTR of the targets. In conclusion, the current study investigated the complexity of the mammary gland transcriptome in dairy cattle using small RNA-seq. Comprehensive analysis of differential miRNAs expression and the data from previous study RNA-seq provided the opportunity to identify the key candidate genes for milk composition traits.

## Background

MicroRNAs (miRNAs), which are a class of non-coding small RNA (sRNA) molecules with the length of 18-24 nucleotides, are important regulators of gene expression. They can play important roles in a wide range of biological processes, including animal and plant development, cell differentiation, proliferation, apoptosis, and metabolism (Martello et al., 2010; Chen et al., 2012; Rottiers and Näär, 2012; Almughlliq et al., 2019; Barbu et al., 2020). In animal cells, miRNAs interact with a specific sequence in mRNA of the target gene and post-transcriptionally negatively regulate the expression of target genes by inhibiting their translation or inducing degradation of the target mRNAs (Huntzinger and Izaurralde, 2011; Barbu et al., 2020). MiRNAs have emerged as new potential biomarkers for miRNA-gene interactions and gene networks responsible for human diseases and economically important traits in livestock. Several diseases and conditions have been reported to be linked with the abnormal expression in miRNAs relating with differentiation, apoptosis and development (Lewis et al., 2005; Berezikov et al., 2006b; Lee et al., 2007). Many experimental techniques and computational methods have been developed to identify miRNAs (Aravin and Tuschl, 2005; Berezikov et al., 2006a; Landgraf et al., 2007), and large number of miRNAs have been identified in primates, rodents, birds, fish, and plants (Lagos-Quintana et al., 2003; Chen et al., 2005; Finucane et al., 2008; Glazov et al., 2008).

The bovine mammary gland is a complex organ which grows and develops after calving and is able to produce more than 30,000 kg of milk in a complete lactation cycle (Hennighausen and Robinson, 2005; Muroya et al., 2019). Because of its important functions, the mammary gland, especially mammary epithelial cells, has been used as the target tissue for gene expression profiling in order to identify key genes underlying milk production traits in dairy cattle (Silveri et al., 2006; Bionaz and Loor, 2008, 2011; Bionaz et al., 2012; Zhang et al., 2016; Pu et al., 2017; Cai et al., 2018; Ju et al., 2018; Yang et al., 2018; Billa et al., 2019; Li et al., 2020). However, only a few studies have been reported related to the miRNAs in the bovine mammary gland. A total of 798 mature bovine miRNAs have been deposited in miRBase (Luoreng et al., 2018), Release 22.1 (October 2018) and 55 of them were detected in the mammary gland. Li et al. (2012b) reported 283 known miRNAs and 74 novel miRNAs in the mammary gland of Holstein cows, among which 56 miRNAs were differentially expressed between lactating and non-lactating cows and might be involved in regulating lactation. Shen et al. (2016) identified 292 known miRNAs and 116 novel miRNAs in the bovine mammary epithelial cells, and three of them (bta-miR-33a, bta-miR-152 and bta-miR-224) might be involved in milk fat metabolism. Li et al. (2015) detected 370 known and 341 novel miRNAs in the bovine mammary gland infected with Staphylococcus aureus, and 358 known and 232 novel miRNAs in control group, 77 of which were differentially expressed between infected and healthy Holstein cows. In addition, Le Guillou et al. (2012) found that the overexpression of miR-30b caused a defect in lactation and delayed involution in mouse mammary gland.

In a previous study from our lab (Cui et al., 2014), 31 differentially expressed genes was identified by using RNA sequencing (RNA-Seq) to investigate the mammary gland epithelial tissues of four lactating Holstein cows with extremely high and low milk protein (PP) and fat percentages (FP). The objectives of the present study were to investigate the miRNA expression profiles in the same mammary gland samples that were used in the previous RNA-Seq study to identify known and novel miRNAs, and to perform an analysis of the differentially expressed miRNAs and previously identified genes. Some candidate miRNAs and their target genes that may be involved in milk protein and fat metabolism were identified.

## Materials and Methods

### Animals and Mammary Gland Tissue Samples

In the current study, the mammary gland epithelium samples of four lactating Chinese Holstein cows (**high group** vs. **low group**) same as our previous RNA-Seq experiment (Cui et al., 2014) were used. These four cows were selected from 30,000 Holstein cows in Beijing Sanyuanlvhe Dairy Farming Center, and the average PP and FP were 3.1% (2.7–3.8%) and 3.6% (3.1–4.5%) in this population. In order to keep the environmental factors identical, these four cows in almost the same period of lactation (353, 341, 377, and 325 days) were collected from the same farm possessing a total of 800 Holstein cows. Selected cows were divided into two groups according to the phenotypic values for PP and FP: two cows (**high group**) had high PP (3.6% and 3.8%) and FP (3.9% and 4.5%); the other two cows (**low group**) showed low PP (3.0%, 2.9%) and FP (3.2%, 3.1%).

The cows were killed by electroshock, and then they were bled, skinned, and dismembered in the same slaughterhouse. The rear mammary gland from each individual was harvested within 30 min after slaughtered. White mammary ducts and pink epithelium tissue were clearly observed when the right rear quarter of the mammary gland was cut in half lengthways from the teat and some milk were flowed out. Five pieces of epithelium tissue samples per cow were carefully collected and placed into a clean RNAse-free Eppendorf tube, and then stored in liquid nitrogen for subsequent RNA isolation. All procedures of collecting samples were carried out in strict accordance with the protocol approved by the Animal Welfare Committee of China Agricultural University (Permit Number: DK996). Total RNA was extracted from one piece of mammary gland epithelium samples from each cow and quality was controlled according to the protocols described by Cui et al. (2014). The value of RNA integrity number (RIN) from each sample was above 8.0.

### Small RNA Sample Preparation and Sequencing

The preparation of small RNA library, including quality control and sequencing, was performed by Novogene (Beijing, China). The preparation of library was performed on 3 μg total RNA per sample using an IlluminaTruSeq™Small RNA Sample Preparation Kit (Illumina, San Diego, CA, United States). The samples were indexed using four codes in order to facilitate sequencing of these samples on one flow cell channel. Quality control in library preparation showed that adapter-adapter contamination was <5% and 85% of the sequences were miRNAs. The samples were subsequently sequenced on the Illumina Hiseq2000 platform and 50-bp single-end reads were obtained.

### Sequencing Data Analysis

The sequencing data were obtained in the format of Illumina FASTQ (Illumina). The procedure of data filtering included removing low quality reads, reads containing poly-N stretches, reads with 5′primer contaminants, reads with 3′primers or the insert tag, and reads with poly-A, T, G, or C stretches. Thereafter, the sRNA tags within a certain range (18-30 nt) were retained for the successive steps. The Q20, Q30, and GC-content of the cleaned reads were calculated to evaluate the quality of data. Then, the sRNA tags were mapped to the bovine genome assembly (UMD3.1.66) using Bowtie (Langmead et al., 2009), no mismatches were allowed and the “seed” region size was set at 8 (Gupta et al., 2012; Giurato et al., 2013; Aggarwal et al., 2014; Kuksa et al., 2018). The mapped sRNA tags were aligned to the 798 bovine miRNA precursor sequences in miRBase (Release 22.1) to identify the known miRNA in the sRNA libraries allowing one mismatch. The sRNA tags that matched known miRNAs from species other than bovine may be novel bovine miRNAs, and were predicted the secondary structure, the Dicer cleavage site, and the minimum free energy of the mapped sRNA sequences using the miREvo (Wen et al., 2012) and miRDeep2 (Friedländer et al., 2012) software packages.

The expression of miRNA was measured as counts per million (CPM) using the following formula: normalized expression = mapped read count/total reads × 1000000 (Zhou et al., 2010), and DESeq2 R package (1.8.3) (Anders and Huber, 2010; Trapnell et al., 2013) was used to identify significantly differentially expressed miRNAs between high and low groups of cows. The threshold for differential expression was—log2 (FC)— > 1 and FDR p < 0.05 when using DESeq2 R package for differential expression miRNA analysis so that miRNAs with—log2 (FC)— > 1 and adjusted FDR p < 0.05 were designated as differentially expressed.

Furthermore, two cows in the same group were used to eliminate the background noise of individual-specific transcription by applying a pairwise approach, which enabled acquisition of more relevant data from the two groups.

### Target Prediction, Pathway, and Annotation Analysis

TargetScan 6.2 and MiRanda (Enright et al., 2003) were used to predict putative target genes with the established miRNA seed database and the bovine genome sequence (UMD3.1.66). TargetScan 6.2 predicts targets by searching for the presence of conserved 8mer, 7mer, and 6mer sites that match the seed region of each miRNA. MiRanda predicts targets based on a development of the miRanda algorithm which incorporates current biological knowledge on target rules and on the use of an up-to-date compendium of mammalian miRNAs.

Gene ontology (GO) functional enrichment analysis was used for the candidate target genes of the miRNAs. GOseq with the Wallenius non-central hyper-geometric distribution (Young et al., 2010), which can adjust for the bias in gene length, was implemented for the GO enrichment analysis. Kyoto Encyclopedia of Genes and Genomes (KEGG) (Kanehisa et al., 2008) pathways analysis was performed using KOBAS 2.0 (Mao et al., 2005) software to test the statistical enrichment of the candidate target genes in the KEGG pathways.

### Quantitative Real Time PCR

Expression levels of selected miRNAs were confirmed by quantitative real-time PCR (qRT-PCR) using the DyNAmo SYBR Green PCR kit (Applied Biosystems, Foster City, CA, United States) on a LightCycler480 (Roche Applied Science, Penzberg, Germany). qRT-PCR of target mRNAs was performed using specific miRNA stem-loop primers (Supplementary Table 8) and all reactions were run in triplicate. Relative quantification of miRNA was quantified using the 2^–Δ^ ^Δ^ ^*CT*^ method and normalized against the *U6* gene (ssD0904071006: Guangzhou RiboBio, Guangzhou, China) for each sample.

### Plasmid Construction and Site-Directed Mutagenesis of 3′UTR in Predicted Target Genes

**FIGURE 1:**
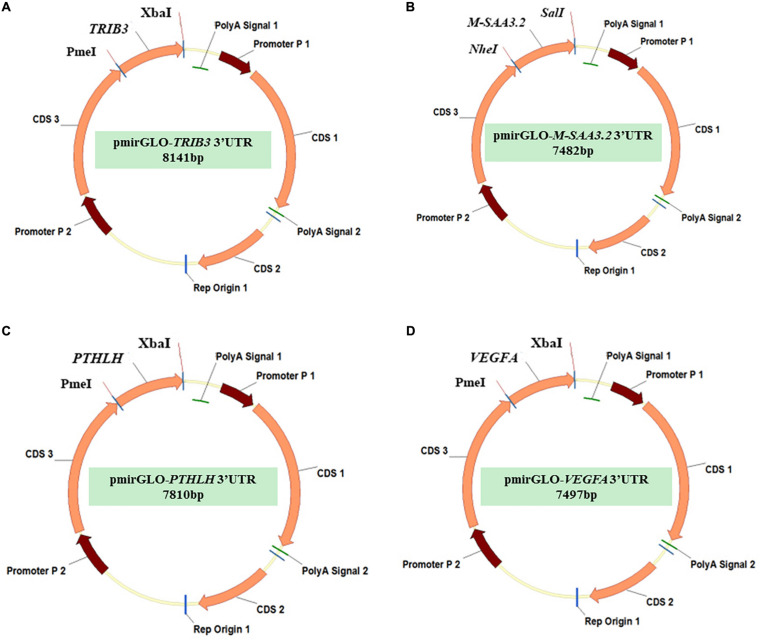
The pmirGLO vectors with the predicted 3’UTR target sequences of the 4 differentially expressed genes **(A)** pmirGLO-*TRIB3*-3′UTR; **(B)** pmirGLO- *M-SAA3.2*-3′UTR; **(C)** pmirGLO-*PTHLH*-3′UTR; **(D)** pmirGLO-*VEGFA*-3′UTR.

**FIGURE 2:**
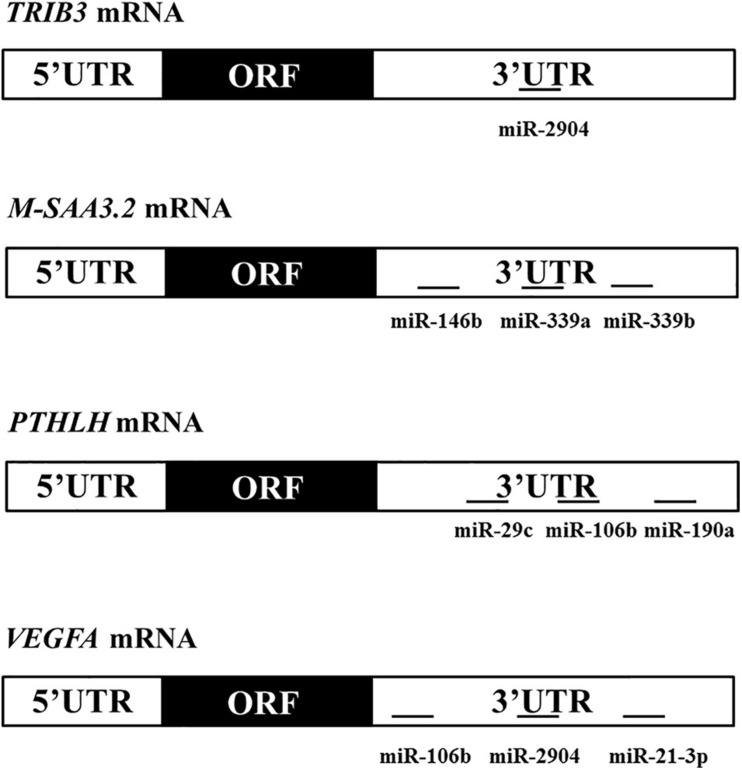
Domain structures of the 4 differentially expressed genes showing the locations of the seed sequence of the miRNAs within the 3’UTR of theirs.

**FIGURE 3:**
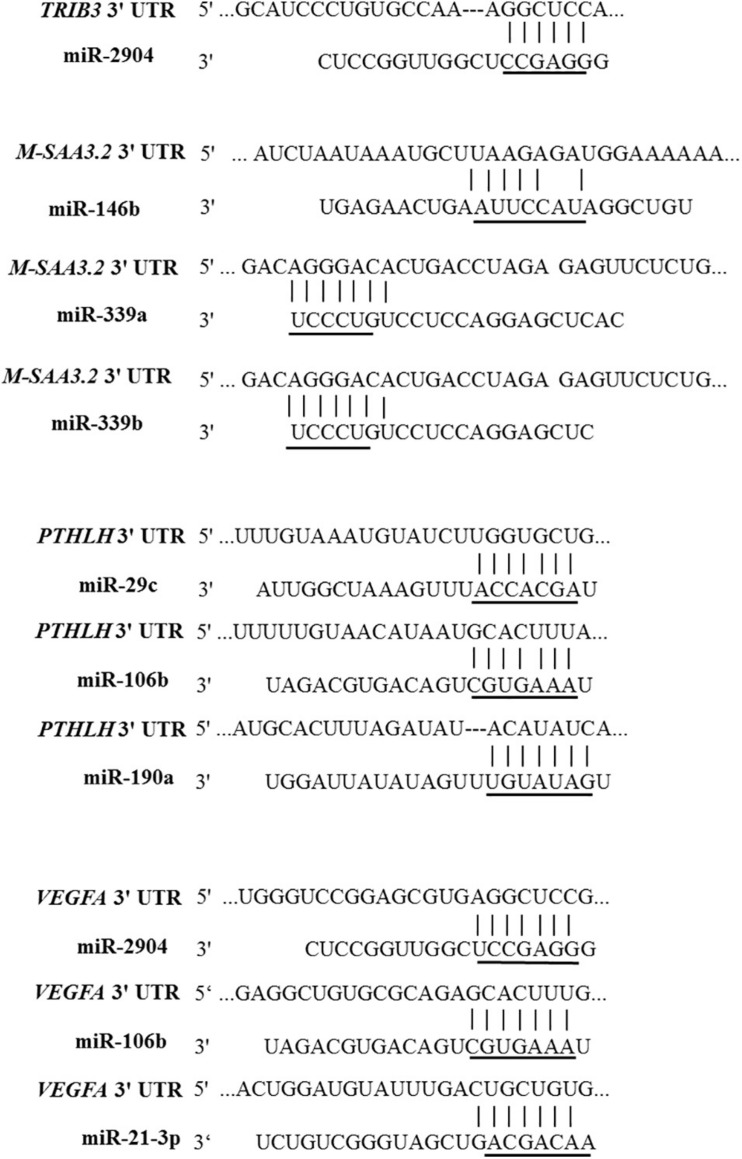
Locations and sequences of the miRNAs target sites in the 3’UTR of the 4 differentially expressed genes. The sequences of the miRNAs are indicated, along with mutations introduced in the target sites (underlined nucleotides) for generating the mutated reporter constructs.

The 3′un-translated region (UTR) of four predicted target genes for the identified miRNAs, *TRIB3*, *M-SAA3.2*, *PTHLH*, and *VEGFA*, were PCR amplified using DNA collected from the bovine mammary gland samples applied for sequencing in this study as a template, and connected into pmirGLO Dual-Luciferase miRNA Target Expression Vector (pmirGLO, Promega) (Figure 1), respectively. The primers were listed in the Table 1. Afterward, the connected products were transfected into Escherichia coli, and then verified the correct sequence and orientation by sequencing. The QuikChange site-directed mutagenesis kit (Stratagene, La Jolla, CA, United States) was used to generate the 3′UTR variants of *TRIB3*, *M-SAA3.2*, *PTHLH*, and *VEGFA* where seed sequences recognized by microRNAs were deleted (Figures 2, 3). After the point mutation, same way was applied in order to find the correct mutant sequences for such four genes.

**TABLE 1:** PCR primers for *TRIB3*, *PTHLH*, *VEGFA* and *M-SAA3.2.*

Gene name	Forward primer sequence	Reverse primer sequence	Amplicon (bp)	Tm (°C)
*TRIB3*	AAAGAGATATGGGTCTCTATGGCTGA	AAGATGGATGAAATATGTAAGAGAGATGACA	806	57
*PTHLH*	TTCTCTTTGCAGGAGGCATTGA	TTCACCTTCTGAGTCATGATGTAATTTAG	475	57
*VEGFA*	AGACGTCTCACCAGGAAAGACT	GACGGAGGTGGGTTAACCACTCA	1050	59
*M-SAA3.2*	GTCATTGATCCCTTGGAAAGAGGAG	CTGTCCTTATACCAAGAATGACACAC	361	59

### Luciferase Reporter Assays

To further explore the repressing mechanism of miRNAs on the expression of 4 target genes (*TRIB3*, *M-SAA3.2*, *PTHLH*, and *VEGFA)* expression, the full-length *TRIB3, M-SAA3.2*, *PTHLH* and *VEGFA* 3′UTRs and the corresponding mutant version (the seed sequences were deleted) were transfected into human embryonic kidney HEK293 cells (GM-070001H: Shanghai, China), respectively. These cells were cultured at 37°C with 5% CO_2_ in Dulbecco’s modified Eagle’s medium (DMEM) supplemented with 4.5 g/liter glucose, 5% fetal bovine serum (Invitrogen), 2 mmol/liter glutamine, and antibiotics. Before transfection, HEK293 cells were plated into 24-well plates at 1.0 × 10^5^ cells/well 24 h. 30 ng empty pmirGLO vector, pmirGLO-*TRIB3/M-SAA3.2/PTHLH/VEGFA*-3′UTR with 50 μl opti-MEM (Invitrogen) and 30 nM (final concentration) mimic miRNA, inhibitor miRNA, control miRNA (GenePharma) were co-transfected into each well with 1 μl Lipofectamine 2000 (Invitrogen). 30 ng mutants of the *TRIB3/M-SAA3.2*/*PTHLH*/*VEGFA* 3′UTR with 50 μl opti-MEM (Invitrogen) and 30 nM (final concentration) mimic miRNA, control miRNA (GenePharma) were co-transfected into each well with 1 μl Lipofectamine 2000 (Invitrogen). Relative firefly luciferase activities (normalized to Renilla luciferase activities) were measured 24 h after transfection with the Dual-Luciferase Reporter Assay Kit (Promega) on TECAN Infinite 200 multifunctional microplate reader (TECAN). All experiments were performed in triplicate so that data averaged from three independent experiments.

## Results

### Sequencing and Mapping of the sRNA Tags

Four new miRNA libraries were constructed using sRNA isolated from bovine mammary glands and sequenced using Illumina next-generation sequencing. A total of 10,538,878 (high milk PP and FP), 12,745,512 (high milk PP and FP), 9,744,027 (low milk PP and FP), and 9,682,136 (low milk PP and FP) high-quality cleaned reads were obtained from the four sRNA libraries (Supplementary Table 1; NCBI SRA accession numbers: SRR3631014, SRR3631016, SRR3631053, and SRR3631054). Distribution of the length for reads showed that most of the generated reads had 21 (>24%), 22 (>30%), and 23 (>13%) nucleotides (Supplementary Figure 1), which is the size of most known mature miRNAs. When aligning the sequenced reads against the bovine genome assembly (UMD3.1.66), it was found that 77.57%, 76.93%, 80.88%, and 78.15% of them uniquely aligned from the four libraries, respectively (Supplementary Table 1); 55-57% of them were aligned in the same direction as the reference genome sequence, and 20-25% were aligned in the opposite direction (Supplementary Table 2). The correlation coefficient (R^2^) between the two individuals within the high and low groups for milk PP and FP was calculated based on the CPM mapped fragment of each cow and was shown to be 0.988 and 0.980, respectively. This indicated that the similarity of the two biological replicates within each group was sufficiently high (Supplementary Figure 2).

### MicroRNAs Identification and Target Prediction

Among the uniquely aligned reads across the four samples and six downloaded miRNA libraries (Cai et al., 2018), 24,320,809 (54.4%) matched known miRNAs in miRBase (Release21.0), which resulted in 497 known bovine miRNAs and 49 novel bovine miRNAs were identified (Supplementary Tables 3, 4). Subsequently, two well-established target prediction tools, TargetScan and miRanda, were used to predict target mRNAs of the miRNAs, and a total of 12,202 target genes were commonly predicted for the known and novel miRNAs (Supplementary Table 5). It is noteworthy that some well-known genes associated with milk composition traits were included such as β-casein (*CSN2*), κ-casein (*CSN3*), α-lactalbumin (*LALBA*), diacylglycerol O-acyltransferase 2 (*DGAT2*), growth hormone receptor (*GHR*), signal transducer and activator of transcription 5B (*STAT5B*), and stearoyl-coenzyme A desaturase (*SCD*) etc. This finding implied that the identified mammary miRNAs in this study were involved in metabolism of milk protein and lipid through the regulation of key genes affecting these traits.

### Differentially Expressed miRNAs Between the High and Low Groups for Milk PP and FP and Target Prediction

The miRNAs that differed between the high and low PP and FP groups were determined in this study. A total of 71 top half miRNAs displayed significantly differential expression between the high and low groups using the DEseq2 algorithm (*p* < 0.05, FDR *q* < 0.05), with 35 were up-regulated and 36 were down-regulated in the high milk PP and FP group compared with the low group (Table 2). Subsequently, a total of 5,634 target genes were commonly obtained for these differentially expressed miRNAs by TargetScan and miRanda (Supplementary Table 7).

**TABLE 2:** Seventy-one differentially expressed miRNAs between the high and low milk protein and fat percentages groups.

miRNA	log2 (fold change)	*p_Value*	*q*_Value	Read counts in high group	Read counts in Low group
miR-21-5p	–1.1183	0	0	503339.5	935032
miR-27a-3p	–1.1042	0	0	60379.5	79260
miR-23a	–1.0206	0	0	44889.5	71388
miR-145	1.0173	0	0	208070.5	147972.5
miR-148a	1.0635	0	0	466750.5	355164.5
miR-143	1.0707	0	0	1175739	785786.5
miR-22-3p	1.2895	0	0	44296.5	28671
miR-3600	1.2895	0	0	44296.5	28671
miR-151-5p	1.3639	0	0	44938.5	27619
miR-10b	1.4156	0	0	103545.5	56277.5
miR-101	–1.1621	0	0	29463.5	45276
miR-100	1.0945	5.50E-289	1.40E-287	55539	48352.5
miR-339a	–1.0197	7.15E-247	1.52E-245	15873.5	23724
miR-191	1.3093	6.74E-233	1.23E-231	34081	25356.5
miR-125b	1.0211	7.42E-228	1.29E-226	46792.5	43191
miR-125a	1.3422	1.60E-185	2.45E-184	22525.5	18141
miR-146b	–1.6573	4.40E-161	9.71E-160	6049.5	8584.5
miR-142-5p	–1.1379	4.30E-147	6.09E-146	9739.5	14961
miR-19b	–1.0332	1.45E-96	2.71E-95	7028.5	10103
miR-29c	–1.0287	1.54E-92	2.76E-91	6245	8204.5
miR-339b	–1.2469	3.98E-87	4.35E-86	3831	5659
miR-10a	1.5514	1.38E-78	1.42E-77	7065	4517
miR-30f	1.0409	3.84E-67	6.78E-66	10956.5	6760
miR-1	2.1527	4.21E-53	3.75E-52	3057.5	1763
miR-221	–1.2284	3.36E-50	5.48E-49	2693	3967.5
miR-106b	–1.2266	3.96E-41	3.16E-40	1653.5	2854.5
miR-142-3p	–1.5988	7.50E-41	5.86E-40	1177.5	2269
miR-34a	–1.1847	1.12E-40	8.58E-40	2131.5	2912
miR-409a	–1.1751	7.26E-40	1.00E-38	2959.5	3838.5
miR-1388-5p	–1.6419	3.96E-32	2.75E-31	908.5	1660
miR-9-3p	–5.2066	9.10E-31	1.12E-29	502	850
miR-9-5p	–2.9879	2.98E-21	3.06E-20	509	750.5
miR-31	–1.1149	4.96E-18	4.56E-17	1507	1851.5
miR-660	1.0526	1.92E-17	1.21E-16	3290	1954.5
miR-19a	–1.2632	2.04E-16	1.66E-15	648.5	1079.5
miR-223	–2.2887	9.68E-16	5.78E-15	585	843.5
miR-133a	2.1331	1.02E-14	5.83E-14	742	366
miR-2904	1.2383	4.05E-14	3.08E-13	1249	719
miR-451	1.1448	1.13E-13	8.33E-13	1690.5	1206.5
miR-215	–1.5896	1.32E-13	7.31E-13	533.5	610.5
miR-2284h-5p	1.1255	3.39E-13	2.41E-12	1491	1039
miR-2285o	1.1255	3.39E-13	2.41E-12	1491	1039
miR-196a	1.5328	4.02E-13	3.30E-12	770	630.5
miR-155	–1.4638	5.45E-13	3.76E-12	640	934
miR-190a	–1.0688	8.23E-10	4.04E-09	502.5	631.5
miR-184	5.1816	1.61E-09	1.17E-08	648.5	519.5
miR-411c-3p	–1.0705	2.31E-09	1.11E-08	535.5	767.5
miR-21-3p	–1.7439	1.36E-08	6.19E-08	551	734.5
miR-136	–1.0645	1.4E-07	5.9E-07	678	854.5
miR-18a	–1.4777	7E-07	2.78E-06	659	780.5
miR-2478	1.2552	9.4E-07	4.99E-06	873.5	606
miR-452	1.0282	0.000017	0.000098	650.5	535
miR-135a	1.2434	0.000017	0.000083	598	522
miR-196b	–1.0123	0.00003	0.00017	795	770.5
miR-345-5p	–1.1028	0.000042	0.00015	591.5	667.5
miR-2887	1.9021	0.000043	0.00015	613.5	500.5
miR-224	1.001	0.00007	0.00037	716.5	536
novel_18	1.7627	0.000084	0.00039	708	574.5
miR-34c	–1.1464	0.000093	0.00032	557.5	559.5
miR-410	–1.1162	0.00011	0.00039	588	676
miR-505	–1.0153	0.00011	0.00053	511.5	608
miR-6522	1.0978	0.00011	0.00058	671.5	524.5
miR-455-5p	–1.4658	0.00012	0.00039	510	569
miR-363	1.1211	0.00019	0.00086	703	548
miR-3431	1.0333	0.00021	0.001	536.5	510.5
miR-331	1.0571	0.00021	0.00067	721	513.5
miR-33a	–1.1294	0.00045	0.00198	549	607
miR-2419-5p	1.2311	0.00058	0.0025	657	556
miR-885	1.3197	0.00179	0.00705	627	585.5
miR-362-3p	–1.0196	1.91E-06	1.03E-05	567	708
miR-378c	1.2476	0.000014	0.000072	675	520

**FIGURE 4:**
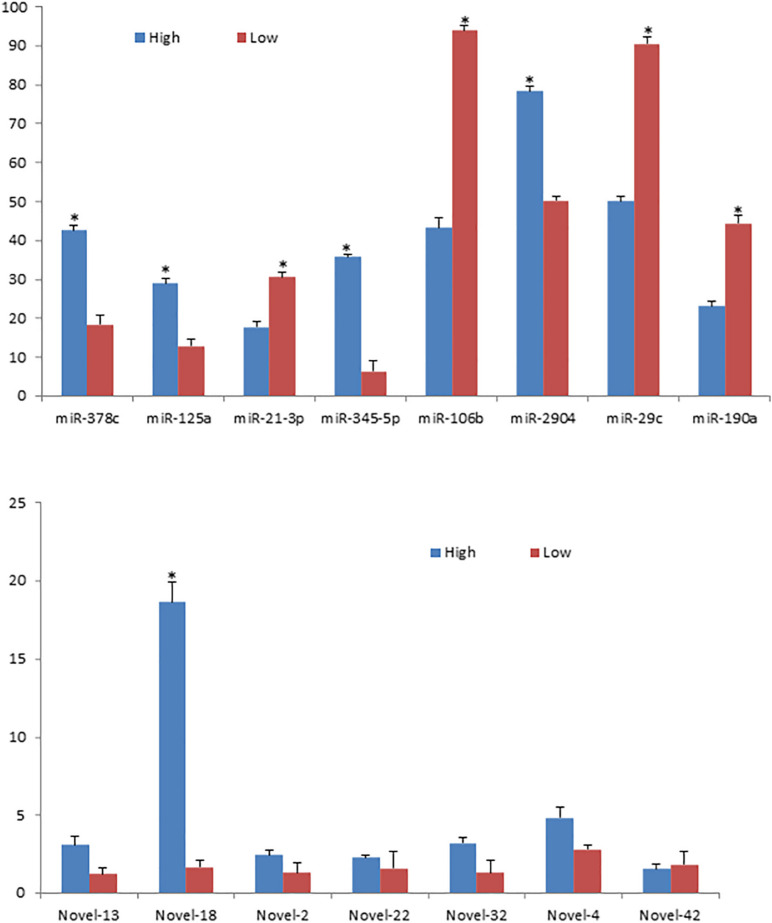
mRNA expression levels of the 15 randomly selected miRNAs validated with qRT-PCR. *indicates *p* < 0.05. Blue columns represent the relative miRNA expression levels by qRT-PCR normalized by *U6* in the high group and red columns represent the relative miRNA expression levels by qRT-PCR normalized by *U6* in the low group.

Afterward, the results of the sequencing were validated with an independent method of real-time PCR assay. By using the same four mammary gland samples as used for sequencing, eight known miRNAs and seven novel miRNAs identified in the present study were randomly chosen for validation. It was found that the expression levels of miR-125a, miR-2904, miR-345-5p, miR-378c and Novel-18 were significantly higher in the high milk PP and FP group than in the low group (*p* < 0.05), and the expression levels of miR-21-3p, miR-29c, miR-106b and miR-190a were lower in the high group than in the low group (*p* < 0.05). Whereas, Novel-13, Novel-2, Novel-22, Novel-32, Novel-4 and Novel-42 did not display significant differences on miRNA levels between the two groups (*p* > 0.05) (Figure 4). Such expression patterns were exactly consistent with those shown by small-RNA sequencing data.

### Gene Ontology Enrichment and Pathway Analysis

To further investigate the functional associations of the target genes, gene ontology (GO) annotation analysis was performed. It was found that these targets have a wide range of diverse functions, among which most were involved in protein and lipid metabolism, mammary gland development and differentiation, and immune functions (*p* < 0.01, FDR *q* < 0.01). Under the GO biological process category, the enriched terms related to lipid and protein metabolisms and cell growth were included such as protein binding, protein localization, protein transport, protein complex, regulation of protein metabolic process, lipid biosynthetic process, programmed cell death, protein targeting, lipid metabolic process, amino acid transport, regulation of protein kinase activity, and cellular response to mechanical stimulus (Supplementary Table 6).

A KEGG metabolic pathway analysis was also performed to identify functions that associate with the predicted target genes using KOBAS. Targets were enriched for functions such as mitogen-activated protein kinases (MAPK), adipocytokine, mammalian target of rapamycin (mTOR), glycosphingolipid biosynthesis, glycerophospholipid metabolism, hypoxia inducible factor-1 (HIF-1), and phosphatidylinositol 3 kinase-protein kinase B (PI3K-Akt) signaling pathways (Table 3).

**TABLE 3:** KEGG pathways assigned to the predicted target genes of the 497 known and 49 novel miRNAs identified in this study.

Pathways	Input number	*p*-Value	Differentially expressed target genes identified in our previous RNA-Seq study^*a,18*^
Lysosome	101	0.00015	
MAPK signaling pathway	182	0.00026	*NR4A1*, *DDIT3*
Endocytosis	182	0.00030	
Leukocyte transendothelial migration	89	0.00119	
Adherens junction	52	0.00166	
Glycosaminoglycan biosynthesis	11	0.00257	
Chagas disease (American trypanosomiasis)	82	0.00260	
mTOR signaling pathway	108	0.00270	*VEGFA*
Synaptic vesicle cycle	47	0.00289	
Adipocytokine signaling pathway	49	0.00299	
Bacterial invasion of epithelial cells	59	0.00299	
Tight junction	102	0.00304	
Collecting duct acid secretion	24	0.00324	
Pertussis	61	0.00327	
Glycosphingolipid biosynthesis	11	0.00345	
SNARE interactions in vesicular transport	30	0.00355	
Glutathione metabolism	44	0.00557	
Homologous recombination	23	0.00774	
Fc gamma R-mediated phagocytosis	68	0.01124	
Linoleic acid metabolism	28	0.02387	
Pyrimidine metabolism	87	0.02475	
Alpha-linolenic acid metabolism	20	0.02550	
Glycerophospholipid metabolism	75	0.03191	
HIF-1 signaling pathway	78	0.03455	*VEGFA*, *CDKN1A*
PI3K-Akt signaling pathway	241	0.03942	*VEGFA*, *CDKN1A*
Apoptosis	22	0.04444	
Arachidonic acid metabolism	55	0.04654	
DNA replication	29	0.04704	

*KOBAS software was used to test the statistical enrichment of the candidate target genes in the KEGG pathways. ^*a*^*NR4A1*, nuclear receptor subfamily 4, group A, member 1; *DDIT3*, DNA-damage-inducible transcript 3; *VEGFA*, vascular endothelial growth factor A; *CDKN1A*,cyclin-dependent kinase inhibitor 1A.*

For the 71 top half differentially expressed miRNAs, 5,634 target genes were obtained and the targets were highly enriched in biological process consisting of synthesis and metabolism of protein and energy metabolism, as well as pathways mainly related to synthesis and metabolism of lipid and protein including glutathione metabolism, NF-kappa B signaling pathway, mTOR signaling pathway, fatty acid degradation, fatty acid metabolism and protein processing in endoplasmic reticulum (Table 4).

**TABLE 4:** KEGG pathways assigned to the predicted target genes of the 71 differentially expressed miRNAs identified in this study.

Pathways	Input number	*p*-Value	Differentially expressed genes identified in our previous RNA-Seq study^*a,20*^
Glutathione metabolism	27	0.015447	
p53 signaling pathway	33	0.01608	*CDKN1A*
Synaptic vesicle cycle	30	0.020395	*CDKN1A*
Cell cycle	51	0.027481	
NF-kappa B signaling pathway	39	0.030627	
Fc gamma R-mediated phagocytosis	37	0.035842	
Collecting duct acid secretion	15	0.047867	*CDKN1A*
mTOR signaling pathway	51	0.027261	*VEGFA*
Fatty acid degradation	15	0.03017	
Fatty acid metabolism	18	0.030625	*DDIT3*
Protein processing in endoplasmic reticulum	49	0.040795	

*KOBAS software was used to test the statistical enrichment of the candidate target genes in the KEGG pathways. ^*a*^*NR4A1*, nuclear receptor subfamily 4, group A, member 1; *DDIT3*, DNA-damage-inducible transcript 3; *VEGFA*, vascular endothelial growth factor A; *CDKN1A*,cyclin-dependent kinase inhibitor 1A.*

### Comparison of the Target Genes of the Differentially Expressed miRNAs and the Differentially Expressed Genes Reported Previously

In our previous study (Cui et al., 2014), 21 of the target genes, which are listed in Table 5, were found to be differentially expressed between the high and low groups using the same four mammary gland samples in the current study. Among the 21 differentially expressed target genes, the expressions of only six down-regulated genes and one up-regulated gene matched the expression profiles of the differentially expressed miRNAs that targeted them. While 5 down-regulated genes were targeted by at least one up-regulated miRNA each, and 10 genes were targeted by both up-regulated and down-regulated miRNAs. Especially, 7 of the 21 differentially expressed target genes were the most promising candidate genes affecting milk protein and fat percentage identified by integrated analysis of differential gene expression, previously reported quantitative trait loci (QTLs) and genome-wide association studies (GWAS) (Cui et al., 2014), including tribbles homolog 3 (*TRIB3*), serum amyloid A1 (*SAA1*), serum amyloid A3 (*SAA3*), mammary serum amyloid A3 (*M-SAA3.2*), vascular endothelial growth factor A (*VEGFA*), parathyroid hormone-like hormone (*PTHLH*) and ribosomal protein L23A (*RPL23A*). In addition, KEGG pathway analysis using KOBAS, showed that two of the 21 target genes, DNA-damage-inducible transcript 3 (*DDIT3*) and nuclear receptor subfamily 4, group A, member 1 (*NR4A1*), were involved in the MAPK signaling pathway that plays critical role in protein synthesis and metabolism and fatty acid metabolism pathway (*p* < 0.05; Tables 3, 4), and 2 other genes, vascular endothelial growth factor A (*VEGFA*) and cyclin-dependent kinase inhibitor 1A (*CDKN1A*), were involved in the mTOR, HIF-1, PI3K-Akt, p53 and duct acid secretion signaling pathways, which are mostly related to synthesis and metabolism of protein and fat (*p* < 0.05; Tables 3, 4).

**TABLE 5:** Twenty-one differentially expressed target genes from our previous study for the 71 differentially expressed miRNAs.

Differentially expressed miRNA identified in this study	log2 (Fold_Change)	*p*-Value	*q*-Value	Differentially expressed target genes identified in our previous RNA-seq study^20^	log2 (Fold_Change)	*p*-Value		*q*-Value
miR-133a	2.1331	1.0203E-14	5.8256E-14	*TRIB3*	–2.64	3.38E-08	2.63E-05	
miR-1388-5p	–1.6419	3.96E-32	2.75E-31					
miR-2904	1.2383	4.05E-14	3.08E-13					
miR-345-5p	–1.1028	0.0000415	0.0001496					
miR-362-3p	–1.0196	1.9053E-06	1.0284E-05					
miR-106b	–1.2266	3.96E-41	3.16E-40	*PTHLH*	–0.76	1.69E-05	0.006194	
miR-190a	–1.0688	8.23E-10	4.04E-09					
miR-29c	–1.0287	1.5384E-92	2.7568E-91					
miR-106b	–1.2266	3.96E-41	3.16E-40	*VEGFA*	–1.25	1.35E-06	0.000647	
miR-125a	1.3422	1.599E-185	2.447E-184					
miR-125b	1.0211	7.422E-228	1.291E-226					
miR-21-3p	–1.7439	1.36E-08	6.19E-08					
miR-2904	1.2383	4.05E-14	3.08E-13					
miR-125a	1.3422	1.599E-185	2.447E-184	*SAA1*	–5.84	9.99E-07	0.000541	
miR-125b	1.0211	7.422E-228	1.291E-226					
miR-146b	–1.6573	4.4E-161	9.71E-160					
miR-146b	–1.6573	4.4E-161	9.71E-160	*SAA3*	–2.09	9.90E-11	1.47E-07	
miR-146b	–1.6573	4.4E-161	9.71E-160	*M-SAA3.2*	–2.49	4.39E-05	0.013339	
miR-339a	–1.0197	7.15E-247	1.52E-245					
miR-339b	–1.2469	3.98E-87	4.35E-86					
miR-378c	1.2476	0.0000142	0.000072	*RPL23A*	–5.31	1.88E-05	0.038988	
miR-135a	1.2434	0.000017	0.0000832	*ATF3*	–2.70	1.24E-06	0.000641	
miR-142-3p	–1.5988	7.5E-41	5.86E-40					
miR-155	–1.4638	5.45E-13	3.76E-12					
miR-21-3p	–1.7439	1.36E-08	6.19E-08					
miR-142-3p	–1.5988	7.5E-41	5.86E-40	*CHAC1*	–2.86	8.62E-08	5.97E-05	
miR-223	–2.2887	9.68E-16	5.78E-15					
miR-339a	–1.0197	7.15E-247	1.52E-245					
miR-339b	–1.2469	3.98E-87	4.35E-86					
miR-106b	–1.2266	3.96E-41	3.16E-40	*SLC25A38*	–0.70	1.57E-07	7.35E-05	
miR-142-5p	–1.1379	4.3E-147	6.09E-146					
miR-143	1.0707	0	0					
miR-224	1.001	0.0000703	0.00037589					
miR-2478	1.2552	9.38E-07	0.00000499					
miR-2904	1.2383	4.05E-14	3.08E-13					
miR-345-5p	–1.1028	0.0000415	0.0001496					
miR-21-3p	–1.7439	1.36E-08	6.19E-08	*NR4A1*	2.42	4.10E-07	0.000243	
miR-224	1.001	0.0000703	0.00037589					
miR-3600	1.2895	0	0	*CDH16*	–1.27	1.29E-06	0.000641	
miR-362-3p	–1.0196	1.9053E-06	1.0284E-05	*EIF4G3*	–0.49	9.76E-06	0.004052	
miR-106b	–1.2266	3.96E-41	3.16E-40	*CDKN1A*	–2.20	1.22E-05	0.004742	
miR-125a	1.3422	1.599E-185	2.447E-184					
miR-125b	1.0211	7.422E-228	1.291E-226					
miR-22-3p	1.2895	0	0					
miR-31	–1.1149	4.96E-18	4.56E-17					
miR-3431	1.0333	0.00021287	0.0010055					
miR-345-5p	–1.1028	0.0000415	0.0001496					
miR-148a	1.0635	0	0	*BOLA-DQB*	–6.92	1.21E-05	0.004742	
miR-106b	–1.2266	3.96E-41	3.16E-40	*H4*	–2.17	2.07E-05	0.007179	
miR-125a	1.3422	1.599E-185	2.447E-184	*FAM71A*	–1.00	2.53E-05	0.008504	
miR-125b	1.0211	7.422E-228	1.291E-226					
miR-224	1.001	0.0000703	0.00037589	*DDIT3*	–1.70	4.01E-05	0.012494	
miR-411c-3p	–1.0705	2.31E-09	1.11E-08					
miR-3431	1.0333	0.00021287	0.0010055	*HIST1H2AC*	–2.01	5.54E-05	0.016423	
miR-363	1.1211	0.00018844	0.0008567					
miR-1388-5p	–1.6419	3.96E-32	2.75E-31	*P4HA2*	–0.69	9.24E-05	0.025587	
miR-23a	–1.0206	0	0					
miR-30f	1.0409	3.84E-67	6.78E-66					
miR-345-5p	–1.1028	0.0000415	0.0001496					
miR-9-5p	–2.9879	2.98E-21	3.06E-20					
miR-190a	–1.0688	8.23E-10	4.04E-09	*C4BPA*	–1.57	9.20E-10	1.04E-06	

*TRIB3, tribbles homolog 3; *PTHLH*, parathyroid hormone-like hormone; *VEGFA*, vascular endothelial growth factor A;*RPL23A*, ribosomal protein L23a; *ATF3*, activating transcription factor 3; *SAA1*, serum amyloid A1; *CHAC1*, cation transport regulator homolog 1; *SAA3*, serum amyloid A3; *SLC25A38*, solute carrier family 25, member 38; *NR4A1*,nuclear receptor subfamily 4, group A, member 1;*CDH16*, cadherin 16; *EIF4G3*, eukaryotic translation initiation factor 4 gamma, 3; *CDKN1A*,cyclin-dependent kinase inhibitor 1A;*BOLA-DQB*, major histocompatibility complex, class II, DQ beta; *H4*, histone H4; *FAM71A*, family with sequence similarity 71, member A; *DDIT3*,DNA-damage-inducible transcript 3;*M-SAA3.2*, mammary serum amyloid A3.2; *HISTH2AC*, histone cluster 1, H2ac;*P4HA2*, prolyl 4-hydroxylase, alpha polypeptide II;*C4BPA*, complement component 4 binding protein, alpha.*

### MicroRNAs Repress the Expression of Target Genes Through the Binding of a Specific Target Sequence in Their mRNA 3′UTR

**FIGURE 5:**
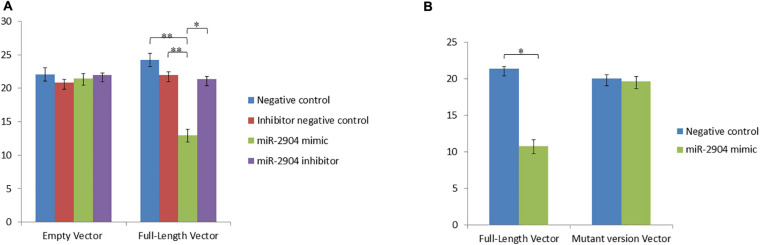
MicroRNAs represses the expression of *TRIB3* via binding the 3’UTR target sequence. Luciferase activity in HEK293 cells co-transfected with miRNA mimic, miRNA inhibitor, miRNA control and empty vector for the *TRIB3* 3’UTR. Luciferase activity was assayed 24 h after transfection. All luciferase values were normalized to Renilla luciferase. Blue columns represent the luciferase activity co-transfected with miRNA mimic control; Red columns represent the luciferase activity co-transfected with miRNA inhibitor control; Green columns represent the luciferase activity co-transfected with miRNA mimic; Pink columns represent the luciferase activity co-transfected with miRNA inhibitor. **(A)** Represents the luciferase activity of *TRIB3* after over- or down-expressed miR-2904 compared with controls. **(B)** Represents the luciferase activity of *TRIB3* after transfecting mutant vector of miR-2904 compared with control. *Significant difference between the control and the treatment; **Very significant difference between the control and the treatment.

**FIGURE 6:**
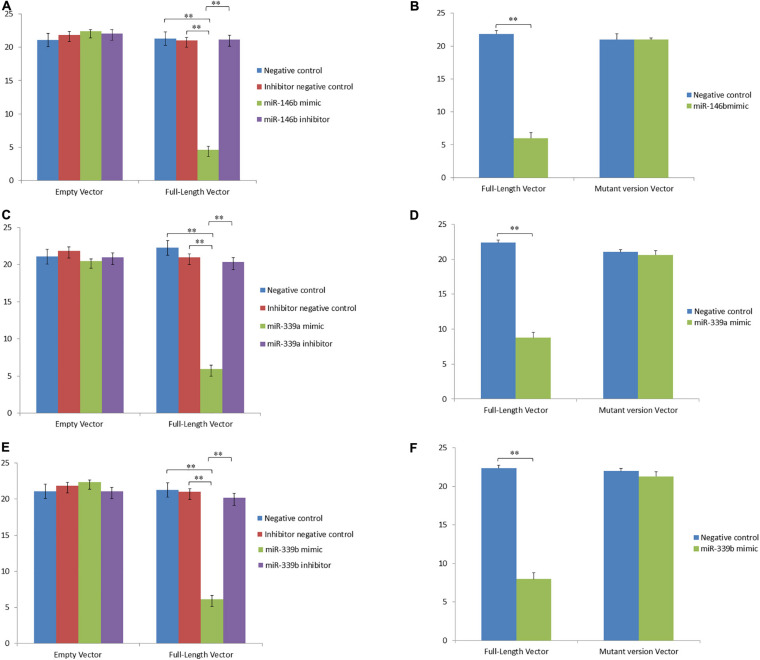
MicroRNAs represses the expression of *M-SAA3.2* via binding the 3’UTR target sequence. Luciferase activity in HEK293 cells co-transfected with miRNA mimic, miRNA inhibitor, miRNA control and empty vector for the *M-SAA3.2* 3’UTR. Luciferase activity was assayed 24 h after transfection. All luciferase values were normalized to Renilla luciferase. The meanings of different colors are consistent with Figure 5. **(A,C,E)** Represents the luciferase activity of *M-SAA3.2* after over- or down-expressed miR-146b, miR-339a and miR-339b compared with controls, respectively. **(B,D,F)** Represents the luciferase activity of *M-SAA3.2* after transfecting mutant vector of miR-146b, miR-339a and miR-339b compared with control, respectively. **Very significant difference between the control and the treatment.

**FIGURE 7:**
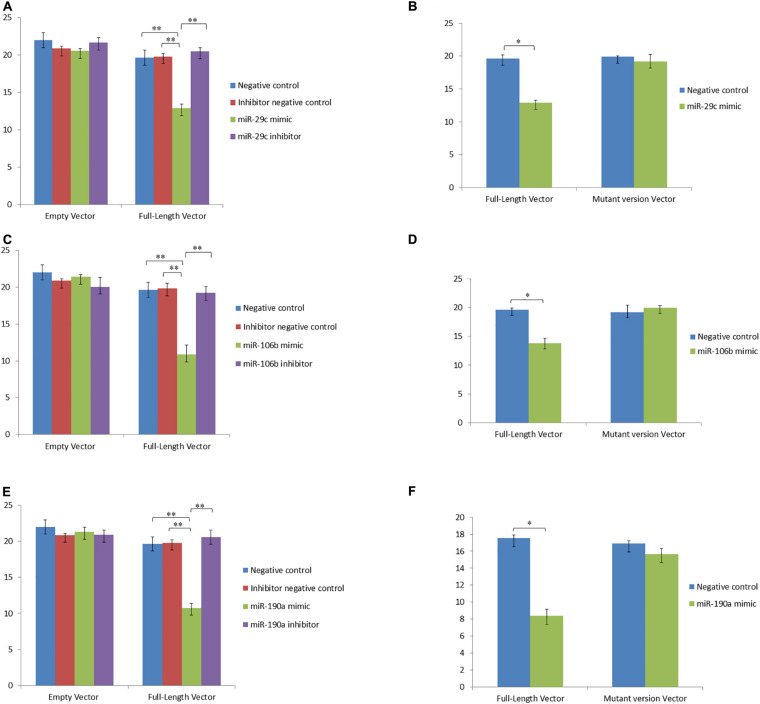
MicroRNAs represses the expression of *PTHLH* via binding the 3’UTR target sequence. Luciferase activity in HEK293 cells co-transfected with miRNA mimic, miRNA inhibitor, miRNA control and empty vector for the *PTHLH* 3’UTR. Luciferase activity was assayed 24 h after transfection. All luciferase values were normalized to Renilla luciferase. The meanings of different colors are consistent with Figure 5. **(A,C,E)** Represents the luciferase activity of *PTHLH* after over- or down-expressed miR-29c, miR-106b and miR-190a compared with controls, respectively. **(B,D,F)** Represents the luciferase activity of *PTHLH* after transfecting mutant vector of miR-29c, miR-106b and miR-190a compared with control, respectively. **Very significant difference between the control and the treatment.

**FIGURE 8:**
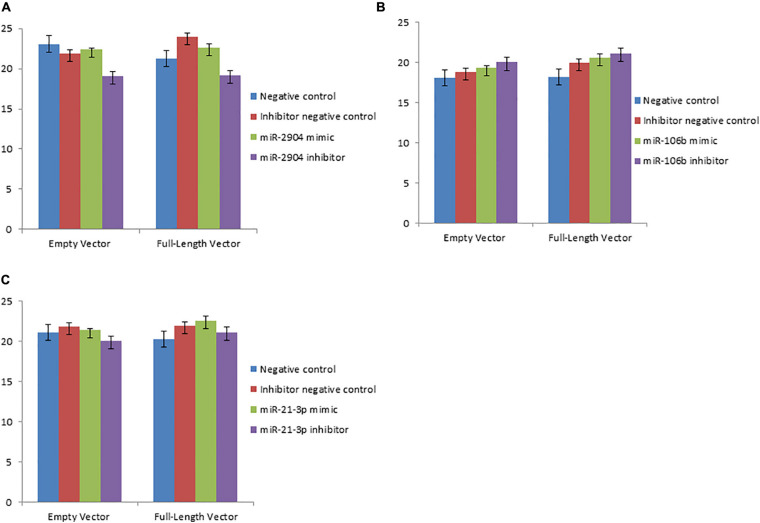
MicroRNAs did not repress the expression of *VEGFA* via binding the 3’UTR target sequence. Luciferase activity in HEK293 cells co-transfected with miRNA mimic, miRNA inhibitor, miRNA control and empty vector for the *VEGFA* 3’UTR. Luciferase activity was assayed 24 h after transfection. All luciferase values were normalized to Renilla luciferase. The meanings of different colors are consistent with Figure 5. **(A,B,C)** Represents the luciferase activity of *VEGFA* after over- or down-expressed miR-2904, miR-106b and miR-21-3p compared with controls, respectively.

To study the regulatory functions of the identified miRNAs, four differentially expressed genes were chosen including *TRIB3*, *M-SAA3.2*, *PTHLH* and *VEGFA*, which having the expression pattern negatively correlated with their targeting miRNAs. Using the dual luciferase reporter assays, whether miR-2904, miR-339b/miR-146b/miR-339a, miR-29c/miR-106b/miR-190a, and miR-2904/miR-106b/miR-21-3p regulated the expression of the *TRIB3*, *M-SAA3.2*, *PTHLH* and *VEGFA*, respectively, were detected. Consequently, it was found that the luciferase level in HEK293 cells with mimics of miR-2904 decreased 40% relative to those with the empty vector, respectively (*p* < 0.05), while the inhibitor of miR-2904 yielded the same luciferase level as negative control (*p* > 0.05) (Figure 5). However, when the predicted binding sites of such miRNA seed sequences were mutated, luciferase activity was efficiently restored to the control levels (*p* > 0.05; Figure 5). Such results clearly indicated the notable regulatory role of the miR-2904 on the expression of *TRIB3* by directly targeting its 3′UTR. Similarly, with regard to *M-SAA3.2*, it was also found that the overexpression of miR-146b, miR-339a and miR-339b decreased the luciferase levels in HEK293 cells by 80%, 72% and 74% after transfecting these mimics compared with the negative controls, respectively (*p* < 0.05), and the depressed expression of such miRNAs did not change the luciferase level in HEK293 cells transfected with their inhibitors (*p* > 0.05), respectively (Figure 6). When the mutant 3′UTR of *M-SAA3.2* and mimics of the 3 miRNAs were co-transfected, the luciferase activity was same as the control level (*p* > 0.05; Figure 6). For *PTHLH*, the luciferase level in HEK293 cells transfected with the mimics of miR-29c, miR-106b and miR-190a was decreased by 37%, 49%, and 50% relative to the negative control, respectively (*p* < 0.05; Figure 7), however, the same level was kept by transfecting the inhibitors of such miRNAs (*p* > 0.05), respectively (Figure 7). When the mutant version of the *PTHLH* 3′UTR and mimics of miR-29c, miR-106b and miR-190a were co-transfected, respectively, the luciferase activities were same as the control levels (*p* > 0.05; Figure 7). Whereas, the expression of *VEGFA* was not affected by miR-2904, miR-106b and miR-21-3p (*p* > 0.05, Figure 8).

## Discussion

The current study is the first comparative profiles of the mRNA and miRNA transcriptome in the mammary gland epithelium of dairy cows to the best of our knowledge. In this study, we generated an extensive miRNA expression profile of the mammary glands from lactating cows with extremely high and low milk PP and FP, and identified a total of 497 known bovine miRNAs and 49 novel bovine miRNAs. In previous studies, bovine miRNAs were identified using computational and direct cloning approaches (Coutinho et al., 2007; Gu et al., 2007; Jin et al., 2009, 2010; Long and Chen, 2009; Li et al., 2012b, 2015; Shen et al., 2016). Li et al. (2012b) identified 298 known miRNAs in lactating and non-lactating mammary gland of Holstein cows using miRNA-seq; 204 of them were among the 497 known miRNAs identified in the current study. Furthermore, 9 of the 71 differentially expressed miRNAs (miR-100, miR-10a, miR-133a, miR-1, miR-146b, miR-148a, miR-221, miR-30f, and miR-339b) identified in the current study were also reported by Li et al. (2012b) as differentially expressed between lactating and non-lactating bovine mammary glands. Gu et al. (2007) identified 31 distinct miRNAs in the mammary glands of Holstein cows, and all of these miRNAs was detected in the present study except miR-142b. Shen et al. (2016) identified 292 known miRNAs in the bovine primary mammary cells, among which 217 miRNAs and 38 differentially expressed miRNAs were also identified in the current study. For the 30 differentially expressed miRNAs in the lactating goat mammary gland fed *ad libitum* or deprived of food affecting milk composition reported by Mobuchon et al. (2015), only 6 miRNAs, including miR-660-5p, miR-451-5p, miR-125b, miR-196a, miR-223-3p, and miR-223-5p were detected as well in the current study. miR-30b related to lactation in mouse (Le Guillou et al., 2012) was also detected in this study, but did not show differential expression between high and low groups. The reason could be due to the mammary gland tissues were collected from different time points of lactation between the previous (Le Guillou et al., 2012) and the current studies.

miR-15a has been reported to be critical in cell development (Bonci et al., 2008), cell cycle (Bandi et al., 2009), and death (Cimmino et al., 2005; Aqeilan et al., 2010). Li et al. (2012a) found that miR-15a can inhibit the viability of mammary epithelial cells as well as the mRNA and protein expression of *GHR*, which is a major gene for milk composition traits (Bonci et al., 2008). In the current study, we also detected miR-15a and predicted that it may target *GHR* as well as candidate genes for milk PP and FP identified in our previous study, namely activating transcription factor 3 (*ATF3*), *VEGFA*, parathyroid hormone-like hormone (*PTHLH*), cation transport regulator homolog 1 (*CHAC1*), and *NR4A1*. Therefore, miR-15a was considered may affect milk composition by regulating the expression of these genes, although miR-15a was not one of the differentially expressed miRNA identified in this study. It was reported that miR-23b inhibited the expression of the transforming growth factor-beta (TGF-β) signaling (Finnerty et al., 2010). In the current study, miR-23b and 5 other miRNAs (miR-2454-3p, miR-496, miR-503-3p, miR-6520, and novel-6) were predicted to regulate *STAT5B*, which is known to be involved in TGF-β signaling (Passerini et al., 2008; Hosui et al., 2009). In addition, genes that are known to affect milk traits (*CSN3*, *CSN2*, *LALBA*, *DGAT2*, *STAT5B*, and *SCD*) were predicted to be targets of some of the identified miRNAs, which implied that they may play critical regulatory roles in mammary gland development and milk composition.

It was found that 21 of 31 differentially expressed genes detected in our previous study (Cui et al., 2014) were the predicted targets for some of the 71 differentially expressed miRNAs detected in the present study. Serum amyloid A1 (*SAA1*), serum amyloid A1 (*SAA3*), and mammary serum amyloid A3.2 (*M-SAA3.2*) were predicted to be regulated by miR-146b (*SAA1* was also regulated by miR-125a and miR-125b); *VEGFA* was regulated by miR-125a, miR-125b, miR-106b, and miR-2904; and ribosomal protein L23a(*RPL23A*), tribbles homolog 3 (*TRIB3*), and *PTHLH* were regulated by miR-378c, miR-2904, and miR-106b, respectively. Moreover, Cai et al., performed RNA sequencing with mammary gland tissue samples from six Chinese Holstein cows with three extremely high and three low milk protein percentage phenotypes and miR-2904, miR-339b, miR-146b, miR-339a, miR-29c, miR-106b, miR-190a, miR-21-3p, miR-15a, miR-486, miR-135, miR-101a, miR-152 and miR-139 were found differentially expressed, which were also identified in our study and targeted on four differentially expressed genes (*TRIB3*, *PTHLH*, *VEGFA*, and *M-SAA3.2*). These seven genes represent the most promising candidates may affect milk PP and FP in dairy cattle (Cui et al., 2014). Specifically, miR-146b was reported to be involved mainly in leukemia, epidermal growth factor receptor (EGFR), MAPK, and nuclear factor kappa-light-chain-enhancer of activated B cells (NF-κB) signaling pathways (Mathews et al., 2004; Taganov et al., 2006; Xiang et al., 2014). The EGFR and MAPK signaling pathways have been demonstrated to be related to adipocyte differentiation (Devaraj et al., 2009; Gao and Bing, 2011) and the NF-κB pathway controls the DNA transcription protein complexes. In human study, miR-146b was shown to regulate the NF-κB signaling pathway in which breast cancer metastasis suppressor 1 (*BRMS1*) has already been implicated, and inhibited both migration and invasion related to metastasis (Taganov et al., 2006; Xiang et al., 2014). Members of the miR-125 family were reported to be implicated in a variety of carcinomas and other diseases as either repressors or promoters. Sun et al. (2013) found that up-regulated miR-125 significantly inhibited the expression of *VEGFA* both *in vitro* and *in vivo* (Jiang et al., 1997). The miR-125 family was found to be a NF-κB-dependent gene in the study by Kim et al. (2012). miR-378c was shown to be involved in the regulation of *RPL23A*, which plays a critical role in translation and participates in apoptosis, cell division, and differentiation (Wool, 1996; Fang et al., 2012; Knezevic et al., 2012). This is consistent with previous reported study where miR-378c was found associated with apoptosis (Lee et al., 2007; Fang et al., 2012; Knezevic et al., 2012; Wang et al., 2014).

The GO and KEGG pathway analyses indicated that *VEGFA*, *NR4A1*, *DDIT3*, and *CDKN1A* were involved in the MAPK, mTOR, HIF-1, and PI3K-Akt signaling pathways, respectively. These four genes were predicted as target genes for miR-106b, miR-2904, miR-125a(b), miR-21-3p, miR-224, miR-31, miR-345-5p, and miR-3431. mTOR signaling is known as playing a fundamental role in adipogenesis (Laplante and Sabatini, 2009), which is the process that leads to the formation of adipose tissue and the most important energy storage site in mammals. It has been demonstrated that mTORC1 positively regulates the activity of sterol regulatory element binding protein 1 (SREBP1) and peroxisome proliferator-activated receptor gamma (PPARG) (Benmoussa et al., 2020), which are two transcription factors that control the expression of genes encoding proteins involved in lipid and cholesterol homeostasis (Kim and Chen, 2004; Porstmann et al., 2008; Kim et al., 2012). HIF-1 is a heterodimeric transcription factor that increases the phosphorylation of signal transducer and activator of transcription 5A (*STAT5A*) in mammary epithelial cells, and the phosphorylation of *STAT5* is known to play important roles in the regulation of milk protein gene expression and mammary development (Shao and Zhao, 2014; Benmoussa et al., 2020). Several studies have shown that hypoxia causes mammary epithelial disorganization and induces a cancer cell-like phenotype in human mammary epithelial cells (MECs) (Whelan et al., 2010; Whelan and Reginato, 2011; Vaapil et al., 2012). The PI3K-Akt pathway has important functions in mammary gland development and function (Wickenden and Watson, 2010). One of the most important functions of Akt is the regulation of glucose homeostasis and metabolism, particularly in muscle and fat tissues (Enright et al., 2003). Therefore, these miRNAs could play critical roles in regulating formation of milk composition trait.

Considering that microRNAs regulate gene expression by targeting specific sequences in the 3′UTR of their cognate genes (Lewis et al., 2005; Friedman et al., 2009), the regulatory roles of some miRNAs on their predicted targets were verified using dual luciferase report assay transfected with mimics, inhibitors and mutants of seed sequences. The results demonstrated that miR-2904, miR-29c/miR-146b/miR-339a, miR-339b/miR-106b/miR-190a indeed down-regulated the expression of the *TRIB3*, *M-SAA3.2* and *PTHLH*, respectively. The molecular mechanisms of how these miRNAs regulate their targets will be further validated through RNAi and over-expression in bovine mammary epithelial cell lines. In addition, it is generally recognized that miRNAs regulate the expression of target genes by inhibiting their translation or inducing degradation of the target mRNAs in animal cells. However, several predicted target genes were regulated in the same direction of expression as those of the corresponding miRNAs between high and low groups. The reason could be due to either target prediction error of the current commonly used prediction softwares (TargetScan 6.2 and MiRanda) or some unknown biological mechanisms. Actually, target prediction was only the first step for studies on interaction between miRNA and their targets. The miRNAs and targets with reverse expression patterns will be considered as the key components for further validation.

In this study, only two biological replicates, which were the same as in our previous RNA-seq investigation (Cui et al., 2014), were used for each condition due to the availability of mammary gland sample from lactating cows, especially high production ones. In order to minimize false-positive errors and ensure substantial detection power and accuracy, two strategies were applied to detect the differentially expressed miRNAs between milking Holstein cows with high PP and FP and cows with low PP and FP, by controlling the critical influencing factors. Small RNA transcripts were deeply sequenced (9-10G data per transcriptome), and only those differentially expressed miRNAs ranked in the top half of the expressed miRNAs were considered, as suggested by Rapaport et al. (2013), Trapnell et al. (2013). Rapaport et al. (2013) investigated the impact of different sequencing depths and number of replicates on the identification of differentially expressed genes, where the authors demonstrated that with most methods, over 90% of differently expressed genes at the top expression levels could be detected with using two replicates and 5% of the reads (Rapaport et al., 2013; Trapnell et al., 2013). The differentially expressed miRNAs expressed in the bottom half level were eliminated to ensure the power in detection. Although mRNA sequencing data was used in this study, detection of differentially expressed miRNAs is based on same statistical theory and software (Anders and Huber, 2010). However, more biological replications are still preferred and recommended in order to provide broader application (Rapaport et al., 2013; Trapnell et al., 2013). The more replicates are performed, the more the detection power is improved. The potential regulatory roles on target genes from such differentially expressed miRNAs will be validated further by performing more in-depth investigation.

## Conclusion

Using sRNA sequencing, 497 known bovine miRNAs and 49 novel bovine miRNAs were identified in the mammary glands of lactating dairy cows. Among all these miRNAs, 71 were differentially expressed between cows with the high and low milk PP and FP. Combined with our previous RNA-Seq data, 21 differentially expressed genes were predicted as the targets for some of the 71 differentially expressed miRNAs. Biological processes related to protein metabolism, fat metabolism, and mammary gland development were enriched for some of the identified miRNAs, which indicated that they may play critical roles in regulating of milk protein and fat traits in dairy cattle. Expression of the *TRIB3*, *M-SAA3.2* and *PTHLH* were significantly down-regulated by miR-2904, miR-29c/miR-146b/miR-339a and miR-339b/miR-106b/miR-190a through binding the specific target sequences in 3′UTR of these genes, respectively.

## Data Availability Statement

The datasets GENERATED for this study can be found in NCBI SRA Accession Numbers: (SRR3631014, SRR3631016, SRR3631053, and SRR3631054).

## Ethics Statement

All experiments, including all protocols for collection of the mammary gland tissues of experimental individuals and phenotypic observations, were performed in accordance with relevant guidelines and regulations of the Institutional Animal Care and Use Committee (IACUC) at China Agricultural University who have reviewed and approved the experiments. Samples were collected specifically for this study following standard procedures with the full agreement of the Beijing Sanyuanlvhe Dairy Farming Center who owned the animals.

## Author Contributions

XC and MY performed the RNA-related experiments, data analysis, and wrote the manuscript. SZ, CW, and QZ participated in the experimental design and helped interpret the results. XG participated in the interpretation of the results, writing, and revision of the manuscript. DS conceived and designed the experiments, and wrote the manuscript. All authors contributed to the article and approved the submitted version.

## Conflict of Interest

The authors declare that the research was conducted in the absence of any commercial or financial relationships that could be construed as a potential conflict of interest.

## Supplementary Material

Supplementary Figure 1 The length distribution of the reads.

Supplementary Figure 2 Correlation plots of the reads for two groups.

Supplementary Table 1 Statistics of the small RNA sequencing data from four mammary gland samples.

Supplementary Table 2 Annotations of the unique mapped sRNA reads from four mammary gland samples.

Supplementary Table 3 Profile of the known bovine miRNAs.

Supplementary Table 4 Profile of the bovine novel miRNAs.

Supplementary Table 5 Prediction results of target genes for the known and novel miRNAs.

Supplementary Table 6 GO terms assigned to the predicted target genes of the known and novel miRNAs.

Supplementary Table 7 Prediction results of target genes for the 71 differentially expressed miRNAs.

Supplementary Table 8 Fifteen randomly selected miRNAs for q-PCR experiments and their primers.

## Abbreviations

PP: protein percentages
FP: fat percentages
MAPK: mitogen-activated protein kinases
mTOR: mammalian target of rapamycin
HIF-1: hypoxia inducible factor-1
PI3K-Akt: phosphatidylinositol 3 kinase-protein kinase B
IACUC: Institutional Animal Care and Use Committee
RIN: RNA integrity number
CPM: Counts per million
UTR: un-translated region
HEK: human embryonic kidney
DMEM: Dulbecco’s modified Eagle’s medium
FDR: false discovery rate
CSN2: β -casein
CSN3: κ -casein
LALBA: α -lactalbumin
DGAT2: diacylglycerol O-acyltransferase 2
GHR: growth hormone receptor
STAT5B: signal transducer and activator of transcription 5B
SCD: stearoyl-coenzyme A desaturase
QTL: quantitative trait loci
GWAS: genome-wide association studies
TRIB3: tribbles homolog 3
SAA1: serum amyloid A1
SAA3: serum amyloid A3 (*SAA3*)
M-SAA 3.2: mammary serum amyloid A3
VEGFA: vascular endothelial growth factor A
PTHLH: parathyroid hormone-like hormone
RPL23A: ribosomal protein L23A
DDIT3: DNA-damage-inducible transcript 3
NR4A1: nuclear receptor subfamily 4
group A: member 1
VEGFA: vascular endothelial growth factor A
CDKN1A: cyclin-dependent kinase inhibitor 1A
ATF3: activating transcription factor 3
CHAC1: cation transport regulator homolog 1
TGF- β: transforming growth factor-beta
EGFR: epidermal growth factor receptor
NF- κ B: nuclear factor kappa-light-chain-enhancer of activated B cells
BRMS1: breast cancer metastasis suppressor 1
SREBP1: sterol regulatory element binding protein 1
PPARG: peroxisome proliferator-activated receptor gamma
STAT5A: signal transducer and activator of transcription 5A
MEC: mammary epithelial cells.
