# Supplementary figures and images for: Comprehensive MicroRNA Expression Profile of the Mammary Gland in Lactating Dairy Cows With Extremely Different Milk Protein and Fat Percentages
Source: Front Genet. 2020 Dec 3;11:548268. doi: 10.3389/fgene.2020.548268 (PMC7744623; doi:10.3389/fgene.2020.548268)

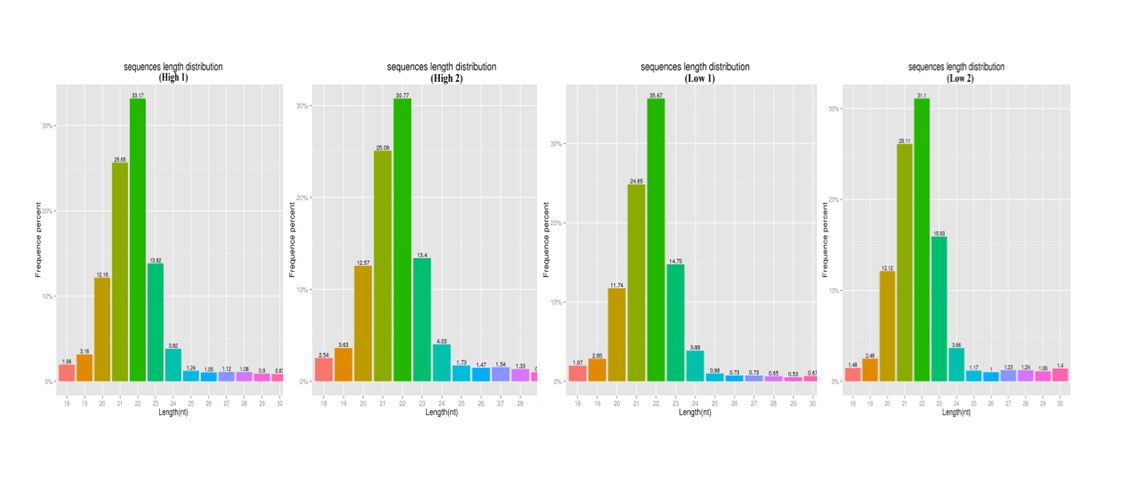

Supplement: Supplementary Figure 1 — The length distribution of the reads. [file Image_1.TIF]

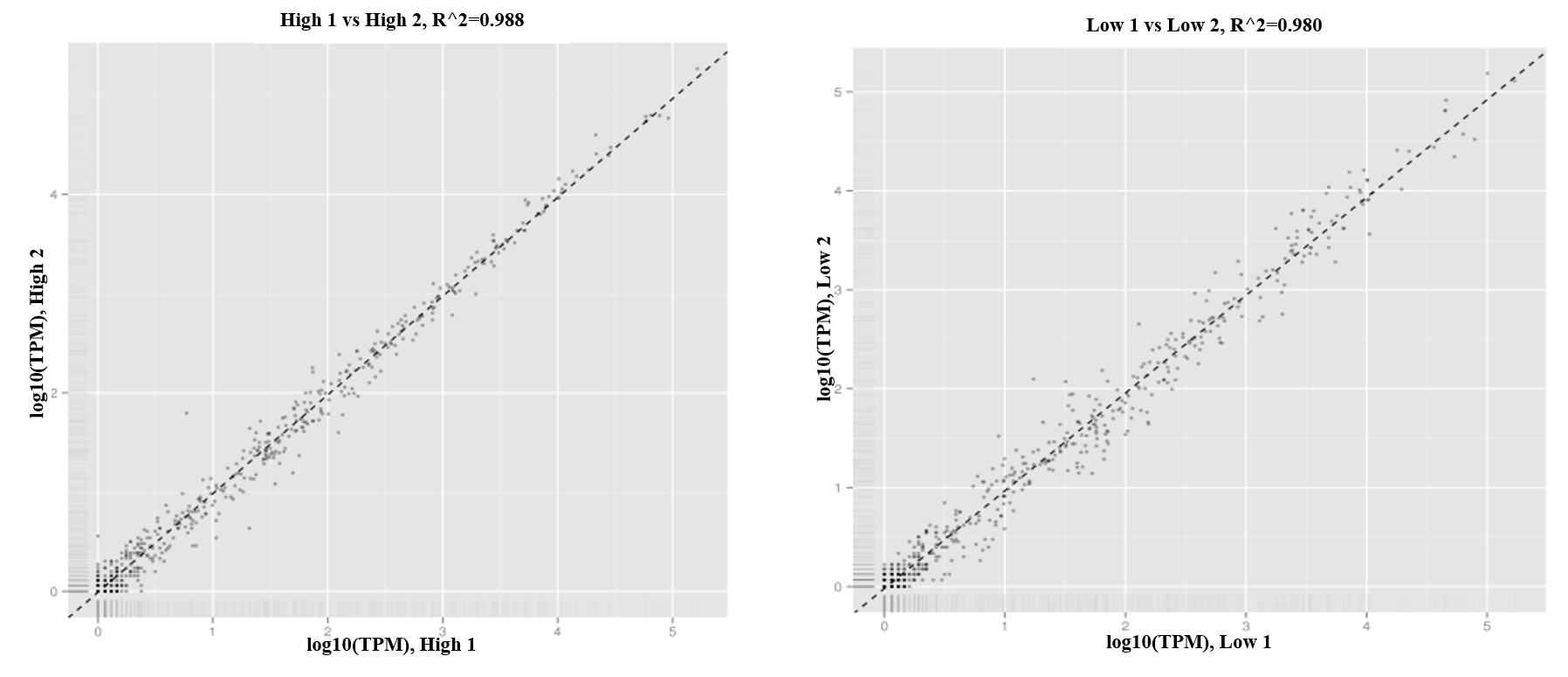

Supplement: Supplementary Figure 2 — Correlation plots of the reads for two groups. [file Image_2.TIF]
